# A simple method to provide positive end expiratory pressure to treat hypoxaemia in an anaesthetised Asian Elephant (*Elephas maximus*)

**DOI:** 10.4102/jsava.v92i0.2118

**Published:** 2021-05-19

**Authors:** Jessica Leung, Thierry Beths, Michael Lynch, Sarah Frith, Sebastien H. Bauquier

**Affiliations:** 1 UVet Animal Hospital, Faculty of Veterinary and Agricultural Sciences, The University of Melbourne, Werribee, Australia; 2 Faculty of Veterinary and Agricultural Sciences, Zoos Victoria, Parkville, Australia

**Keywords:** anaesthesia, Asian elephant, Elephas maximus, hypoxaemia, Positive end-expiratory pressure, ventilation

## Abstract

Hypoxaemia is a common complication in anaesthetised or immobilised elephants. It is presumably because of hypoventilation and ventilation-perfusion mismatch. To prevent hypoxaemia, orotracheal intubation and positive pressure ventilation are recommended. This case report describes a hypoxaemic period despite positive pressure ventilation in a 46-year-old female Asian elephant (*Elephas maximus*) anaesthetised with azaperone-etorphine, medetomidine and an etorphine constant rate infusion in lateral recumbency for a dental procedure. The hypoxaemia was corrected utilising positive end-expiratory pressure (PEEP) of 5 cm – 10 cm H_2_O, a technique that has not previously been reported in the management of anaesthetised elephants. PEEP decreases atelectasis, shunt fraction, and increases lung compliance. Positive end-expiratory pressure was achieved by partial occlusion of the tailpiece of a manually triggered demand valve ventilator during expiration. This is a simple effective method of generating PEEP and correcting hypoxaemia without the need for any additional specialised equipment. However, PEEP decreased arterial blood pressure and should be implemented with caution if arterial blood pressure is not monitored.

## Introduction

Hypoxaemia is a frequently reported complication in captive and free-ranging immobilised elephants (Honeyman, Pettifer & Dyson 1992; Horne & Loomis 2014; Stegmann, Grobler & Zuba 2014). Apart from the use of intermittent positive pressure ventilation (IPPV), there is scarce literature regarding its prevention and correction (Horne & Loomis 2014; Horne, Tchamba & Loomis 2001; Stegmann et al. 2014). This case report describes the use of positive end-expiratory pressure (PEEP) to alleviate hypoxaemia in a laterally recumbent, anaesthetised Asian elephant (*Elephas maximus*).

## Patient presentation

A 46-year-old female *E. maximus* weighing 3340 kg (several months prior to anaesthesia) was anaesthetised for extraction of the right mandibular molar 3. Food had been withheld for 17 h and water withheld for 15 h prior to anaesthesia. The elephant was immobilised with 3 *μ*g/kg etorphine IM (Etorphine, Wildlife Pharmaceuticals, South Africa) and 9 *μ*g/kg azaperone IM (Stresnil, Elanco, Australia) administered by hand injection. Fifteen minutes after initial immobilisation agents were delivered, profound sedation was achieved and the animal was guided into left lateral recumbency onto a deep sand bed using a rope casting technique. The left auricular vein was catheterised and 1.2 *μ*g/kg medetomidine intravenous (IV) (Medetomidine, Ilium, Glendenning, Australia) was administered. Anaesthesia was maintained using an etorphine constant rate infusion (0.6 *μ*g/kg/h – 1.5 *μ*g/kg/h IV). The etorphine was diluted in 1 L of lactated ringers solution and run through a fluid pump to ensure it was delivered at a constant rate. Heart rate (HR), pulse oximetry (SpO_2_), respiratory rate (RR), end-tidal CO_2_, inspired oxygen concentration (FiO_2_), electrocardiography and invasive blood pressure (BP) (18 g catheter in the auricular artery) were monitored using a calibrated multi-parameter anaesthetic monitor (Mindray, Notting Hill, Victoria, Australia). Arterial blood gas tests were performed using a handheld blood gas monitor iStat, Abbott Laboratories, United States (US).

As a result of difficulties with intubation and a period of apnoea that developed after the medetomidine administration, 6 *μ*g/kg atipamezole was administered IV (Atipamezole, Ilium, Australia). Shortly after reversal, a 30-mm internal diameter (ID) cuffed orotracheal tube was placed and connected to a manually triggered Mega-Vertebrate demand ventilator (In Case of Anesthesia, US). With a peak inspiratory pressure (PIP) of 15 cm H_2_O, RR of 7 and an inspiratory: expiratory ratio (I:E) of 1:1, it allowed the maintenance of normocapnia (EtCO_2_ between 35 millimetre of mercury [mmHg] and 45 mmHg), while providing a FiO_2_ of about 33%.

After 1 h into the anaesthetic, the SpO_2_ value decreased from 100% to 92%. A leak around the orotracheal tube was detected and another 30-mm ID tube with a slightly larger external diameter was placed. Although the PIP was increased to 20 cm H_2_O, the SpO_2_ value declined between 86% and 89%. An arterial blood gas analysis confirmed a diagnosis of hypoxaemia with a PaO_2_ of 49 mmHg and a PaCO_2_ of 55 mmHg (see Table 1).

**TABLE 1:** Cardiorespiratory variables over the duration of the anaesthetic for an elephant anaesthetised with etorphine-azaperone, medetomidine and an etorphine constant rate infusion.

Time	Event	HR	RR	SPO_2_	ETCO_2_	MAP	PaO_2_	PaCO_2_
9:07	Etorphine/azaperone injection	-	-	-	-	-	-	-
9:22	Recumbency achieved	-	-	-	-	-	-	-
9:23	Cannulation of auricular vein, 4 mg medetomidine IV	39	5	97	-	-	-	-
9:27	First intubation attempt	42	0	96	-	-	-	-
9:29	Second intubation attempt with smaller ETT	40	0	97	-	-	-	-
9:30	Atipamezole 20 mg IV	42	0	96	-	-	-	-
9:31	Intubation	37	0	99	-	-	-	-
9:32	Commence IPPV	37	6	100	34	-	-	-
9:37	Commence etorphine CRI	32	6	95	30	-	-	-
9:55	Arterial line placement	46	7	100	31	172	78	47.2
10:10	Commence dental procedure	47	7	97	28	170	-	-
10:16	Desaturation	40	7	92	30	138	76	56.8
10:25	Extubate because of leaking ETT	54	-	88	-	132	-	-
10:31	Reintubate with larger ETT	50	7	86	23	120	-	-
10:40	Before start of PEEP	33	7	89	25	91	49	54.9
10:50	Removed arterial line	-	7	94	19	70	59	23.6
10:55	Removed remainder of monitoring	-	-	97	-	-	-	-
11:00	End anaesthesia, administered naltrexone and atipemazole	-	-	-	-	-	-	-
11:02	Standing	-	-	-	-	-	-	-

EtCO_2_, end-tidal carbon dioxide; ETT, endotracheal tube; HR, heart rate; MAP, mean arterial pressure; PaCO_2_, partial pressure of arterial carbon dioxide; PaO_2_, partial pressure of arterial oxygen; RR, respiratory rate; SpO_2_, oxygen saturation; IPPV, intermittent positive pressure ventilation; PEEP, positive end-expiratory pressure; CRI, constant rate infusion.

To treat the hypoxemia, it was decided to add PEEP. At the end of the inspiration phase, the anaesthetist let the air pressure decrease until it reached 5 cm – 10 cm H_2_O. At that time, using his hand, the anaesthetist applied PEEP by blocking the ventilator’s exhaust tailpiece to prevent the air pressure from decreasing further until the next breath. The PIP, RR and the I:E ratio remained unchanged at 20 cm H_2_O, 7 breaths a minute and about 3.5:5, respectively. Within 7 min, the PaO_2_ increased to 59 mmHg (SpO_2_ of 92%), and shortly after, the SpO_2_ reached 97%; however, further blood gas analysis was not available.

While oxygenation improved with PEEP, BP declined from a mean arterial pressure of 132 mmHg immediately before PEEP to as low as 70 mmHg during PEEP. In preparation for recovery, the arterial catheter was removed and PEEP was discontinued because of the risk of unmonitored hypotension.

## Discussion

The potent opioid etorphine is often used to anesthetise elephants either alone or in combination with other drugs (Honeyman et al. 1992; Horne et al. 2001; Stegmann et al. 2014). It often causes hypoventilation and moderate to severe hypoxaemia (Horne & Loomis 2014; Stegmann et al. 2014). In conscious *E. maximus*, lateral recumbency for 15 min is also associated with a significant decrease in PaO_2_ when compared with standing. This reduction in PaO_2_ can be as significant as 30% but is usually not accompanied by any change in PaCO_2_ (Honeyman et al. 1992; Isaza et al. 2003). As such, hypoventilation is hypothesised to contribute less to hypoxaemia in conscious recumbent elephants than ventilation/perfusion (V/Q) mismatch and pulmonary shunting, similar to horses (Horne & Loomis 2014). In the latter, the degree of V/Q mismatch correlates positively with increasing body mass and being influenced by the shape of the thoracoabdominal contour (Moens et al. 1998). Therefore, the physical attributes of elephants, including a high body mass and extremely oblique set diaphragm, may contribute to the rapid formation of V/Q mismatch (Horne & Loomis 2014; Stegmann et al. 2014). The use of etorphine may further exacerbate V/Q mismatch as it is a known cause of pulmonary hypertension in other species (Meyer et al. 2015). Extreme pulmonary hypertension may result in right to left shunt, thus contributing to hypoxaemia.

As a result of the use of potent respiratory depressants and the rapid development of V/Q mismatch, oxygen supplementation is recommended for all immobilised or anaesthetised elephants (Horne & Loomis 2014). Although elephants are primarily nasal breathers, oxygen insufflation via the trunk is usually unsuccessful in preventing hypoxaemia (Horne & Loomis 2014; Stegmann et al. 2014). Intermittent positive pressure ventilation through an orotracheal tube is therefore the preferred method of ventilation with multiple modes of IPPV reported in that species. Such methods of IPPV include the use of modified leaf blowers to deliver room air in the field, high flow demand valves connected in series to deliver 100% oxygen and customised bellow driven ventilators for use in a clinic environment (Horne & Loomis 2014; Horne et al. 2001).

In this case, a combination of drug-induced hypoventilation and the development of V/Q mismatch are the likely causes of the observed hypoxaemia. Correction of hypoventilation by providing IPPV and increasing PIP was unsuccessful in correcting hypoxaemia. In anaesthetised horses, atelectasis forms rapidly, and the early implementation of IPPV is recommended to maintain and improve oxygenation (Moens et al. 1995, 1998; Wilson & Soma 1990). The delay in implementing IPPV because of difficulties in intubation and the initial period of apnoea may have contributed to the development of atelectasis, V/Q mismatch and consequently hypoxaemia in this case. The anaesthetist was unable to increase PIP past 20 cm H_2_O, which did not allow for a proper recruitment manoeuvre and was followed by a further decrease in SpO_2_.

This case utilised a commercially available assisted demand ventilator. The design of this specific ventilator is such that rapid and high flow oxygen delivery into the ventilator generates negative pressure at the tailpiece of the ventilator and draws in atmospheric air through entrainment. The increase of gas flow permits a quicker delivery of the required tidal volume. The resulting FiO_2_ was 33%, although a FiO_2_ of 42% is reported by the manufacturer (In Case of Anaesthesia, n.d.). As high FiO_2_ is associated with increased shunt fraction in other species, the lower FiO_2_ provided by this ventilator may have been beneficial in preventing further atelectasis (De Monte et al. 2013; Osbertg et al. 2017).

Positive end-expiratory pressure, which is defined as the residual alveolar pressure above atmospheric pressure at the end of expiration, improved oxygenation (Ambrosio et al. 2013; Moens et al. 1998). The mechanism by which PEEP achieved this is multifactorial. It includes increased alveolar pressures to reopen alveoli and prevent alveoli collapse, decreased work of breathing, increased functional residual capacity and altering the centre of distribution of ventilation within the lungs (Ambrosio et al. 2013; De Monte et al. 2013).

Positive end-expiratory pressure improves pulmonary gas exchange in multiple species under anaesthesia (Ambrosio et al. 2013, 2017; Osbertg et al. 2017). In humans, a PEEP around 6 cm H_2_O prevents atelectasis, although a higher PEEP is required to reopen closed alveoli (Osbertg et al. 2017). In dogs, a PEEP at 10 cm H_2_O results in a significant reduction of atelectasis and an increase in lung volume by up to 60%, while in horses, titration of the PEEP improves oxygenation, reduces shunt fraction and increases lung compliance (De Monte et al. 2013; Moens et al. 1998).

On a modern mechanical ventilator, PEEP is a basic setting that applies pressure at the end of expiration. Otherwise, commercial PEEP valves or handmade water column can be adapted onto the expiratory limb of the circuit or the scavenge tubing of the anaesthesia machine (Allison et al. 2017). In this case, suitable PEEP valves were not available, and use of a water column was inappropriate because of the risk of water being funnelled into the circuit. Covering the tailpiece of the ventilator at expiration was effective at generating PEEP. This is a simple method of generating PEEP without the need for any specialised equipment.

The use of PEEP in elephants may be of particular use in recruiting collapsed alveoli and improving oxygenation because of their unique respiratory system. Elephants lack a pleural space, instead, they possess a distensible collagen fibre network that adheres to the pleura of the chest wall. In addition, the parenchyma itself is supported by an elastic septum that limits alveoli collapse (Browne et al. 1997). These anatomical features may explain the effective recruitment of alveoli and improved oxygenation when applying low levels of PEEP in this elephant. Additionally, following recruitment of the alveoli, the application of PEEP or the reduction of the FiO_2_ to 40% was shown to improve and maintain oxygenation and respiratory compliance in dogs (De Monte et al. 2013). The combination of PEEP and reduced FiO_2_ provided by the ventilator may have aided the improved oxygenation in this elephant.

As a result of the increased intrathoracic pressures generated by PEEP, cardiac output and consequently, BP often declines (Luecke & Pelosi 2005; Wilson & Soma 1990). This is primarily because of a decrease in venous return and thus ventricular stroke volume (Luecke & Pelosi 2005). In this case, PEEP resulted in a reduction of BP by nearly 50% and thus PEEP was titrated to avoid further decline in BP while maintaining oxygenation. Arterial BP should therefore be monitored closely when PEEP is implemented.

Although the application of PEEP may impair cardiac output, the period of hypotension in this animal may have been exacerbated by other factors. The withholding of water prior to anaesthesia may have produced a mild hypovolaemia resulting in hypotension. In addition, the use of azaperone and atipamezole may have caused vasodilation, thus further reducing BP. The aetiology of the hypotension was likely multifactorial and PEEP impaired the ability of the animal to compensate adequately for its impaired cardiovascular status.

In conclusion, PEEP could easily be generated on the Mega-Vertebrate demand ventilator and was effective at improving oxygenation in laterally recumbent *E. maximus* undergoing general anaesthesia.
